# An outcome measure of functionality in patients with lumber spinal stenosis: a validation study of the Iranian version of Neurogenic Claudication Outcome Score (NCOS)

**DOI:** 10.1186/1471-2377-12-101

**Published:** 2012-09-24

**Authors:** Parisa Azimi, Hassan Reza Mohammadi, Ali Montazeri

**Affiliations:** 1 Department of Neurosurgery, Shahid Beheshti, University of Medical Sciences, Tehran, Iran; 2 Mental Health Research Group, Health Metrics Research Centre, Iranian Institute for Health Sciences Research, ACECR, Tehran, Iran

## Abstract

**Background:**

Neurogenic claudication (NC) is a common symptom in patients with lumbar spinal stenosis (LSS). The Neurogenic Claudication Outcome Score (NCOS) is a very short instrument for measuring functional status in these patients. This study aimed to translate and validate the NCOS in Iran.

**Methods:**

This was a prospective clinical validation study. The 'forward-backward' procedure was applied to translate the NCOS from English into Persian (Iranian language). A total of 84 patients with NC were asked to respond to the questionnaire at two points in time: at preoperative and at postoperative (6 months follow-up) assessments. The Oswestry Disabiltiy Index (ODI) also was completed for patients. To test reliability, the internal consistency was assessed by Cronbach's alpha coefficient. Validity was evaluated using known groups comparison and criterion validity (convergent validity). Internal responsiveness of the NCOS to the clinical intervention (surgery) also was assessed comparing patients’ pre- and postoperative scores.

**Results:**

The Cronbach’s alpha coefficients for the NCOS at preoperative and postoperative assessments were 0.77 and 0.91, respectively. Known groups analysis showed satisfactory results. The instrument discriminated well between sub-groups of patients who differed in claudication distance as measured by the Self-Paced Walking Test (SPWT). The change in the ODI after surgery was strongly correlated with change in the NCOS, lending support to its good convergent validity (r = 0.81; P < 0.001). Further analysis also indicated that the questionnaire was responsive to the clinical intervention (surgery) as expected (P < 0.0001).

**Conclusion:**

In general, the Iranian version of the NCOS performed well and the findings suggest that it is a reliable and valid measure of functionality in patients with lumbar spinal stenosis who are suffering from neurogenic claudication.

## Background

Low back pain is a common musculoskeletal disorder affecting 80% of people at some points in their lives. The term lumbar spinal stenosis (LSS) refers to the anatomic narrowing of the spinal canal in the anterior-posterior axis [1]. Neurogenic claudication (NC) is a common symptom of LSS, which is a term proposed by Dejerine [2] in 1911 and further refined by von Gelderen [3] in 1948. Symptoms of NC are described as pain, paraesthesia or cramping of one or both legs, brought on when walking and relieved in sitting [4,5]. To measure NC, the Neurogenic Claudication Outcome Score (NCOS) was developed by Weiner and Fraser [6]. As suggested it is a simple, concise, self-administered outcome questionnaire and specifically tailored to address functionality in patients with neurogenic claudication [7]. Several studies used the NCOS as an outcome measure of functionality in patients with lumbar spinal stenosis [7-11]. In fact the NCOS is based on and represents an expansion of the Low Back Outcome Score developed by Greenough and Fraser. They did a number of psychometric tests (observer and inter-observer variation analysis, convergent and discriminant validity), and reported that the questionnaire was a reliable and valid measure of functionality in low back patients [12].

The aim of this study was to translate the NCOS from English into Persian (Iranian language), validate and use the questionnaire in studies of functionality in NC patients in Iran. Currently there is no such questionnaire available in Iran.

## Methods

### The questionnaire

**Figure 1:**
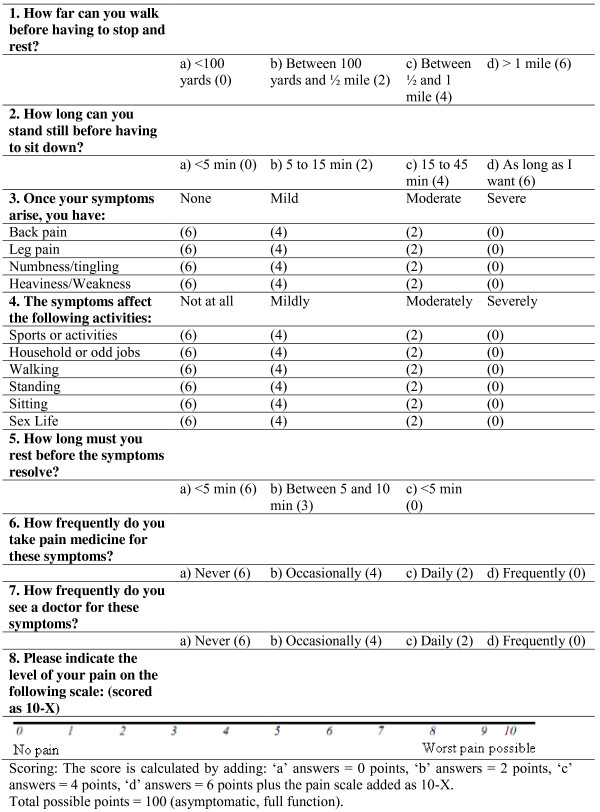
The Neurogenic Claudication Outcome Score (NCOS).

The Neurogenic Claudication Outcome Score (NCOS) is an specific measure of functionality in patients with neurogenic claudication. It consists of 8 questions with some questions containing items related to different functioning (questions 3 and 4), giving a total of 16 items for the questionnaire. Each item is rated on a four-point scale with two-point intervals ranging from 0 to 6 (0-2-4-6) indicating worst to best conditions expect for pain intensity where a 100-mm visual analogue scale is used. Patients select the point on the line that best represents his/her perception of pain intensity. The scale score then is calculated as the sum of all items ranging from 0 to 100 with higher scores indicating higher levels of functioning and/or better health status (Figure 1).

### Translation

The ‘forward-backward’ procedure was applied to translate the NCOS from English into Persian (Iranian language). Two general practitioners translated the questionnaire into Persian. One translator was aware of the project and the other translator was not. Both translators were instructed to aim for conceptual rather than literal translation [13]. Together with the main investigator (PA) the translators compared their translations and produced a single provisional version of the questionnaire. Then, two other professional translators translated the provisional Persian questionnaire back into the English language [14]. Finally, an expert committee consisting of the translators, the researchers, one outcome methodologist reviewed the translation and cultural adaptation processes. After a careful review and cultural adaptation, few changes have been made, and the pre-final Persian version of the questionnaire was produced. Testing of this pre-final version was performed in the following way. A number of patients with NC completed the pre-final Persian version of the NCOS to establish that this version could be understood and that the questions measured what they were intended to measure. For each item patients were asked to respond to the following questions: ‘Do you understand what this means’? and, ‘What does this mean to you by your own words’? Most patients correctly understood the questionnaire and the concept of each item. However, their general comments on difficulty in completing the questionnaire or understanding the texts were examined, and after a consensus by authors the final version was developed and used in this study.

### Patients and data collection

The final draft of the Iranian version was administered to a sample of newly diagnosed NC patients attending the neurosurgery clinic of a large teaching hospital in Tehran, Iran. There were no restrictions on patient selection with regard to severe, moderate and mild NC, age (to include all types of stenosis that are congenital and degenerative), other characteristics, and any grade stenosis as explained by Constantin and colleagues [15]. They described a 7-grade (A: A1, A2, A3, A4, B, C and D) classification of severity of lumbar spinal stenosis based on the morphology of the dural sac as observed on T2 axial magnetic resonance images and based on the rootlet/cerebrospinal fluid ratio. They defined grade A, as no or minor stenosis, B as moderate stenosis, C as severe stenosis, and D as extreme stenosis.

Since there were some illiterate patients, we decided to collect the data by face-to-face interviews. To avoid intra-rater bias, only one of us (PA, a trained neurosurgery resident) interviewed the patients. Patients were assessed at two points in time: at pre-operation and at post-operation (6 moths follow-up). Postoperative assessment was carried out due to the fact that we were interested to evaluate outcomes and perform responsiveness analysis.

### Additional measures

1. The Iranian version of Oswestry Disability Index (ODI): This is a measure of functionality and contains 10 items. The possible score on the ODI ranges from 0 to 50, with higher scores indicating worst conditions. The psychometric properties of Iranian version of ODI are well documented [16]. The questionnaire was used to examine criterion validity.

2. The Self-Paced Walking Test (SPWT): The SPWT is a measure for walking capacity, which is the distance a person with LSS is able to walk without support on a level surface at a self-selected speed before being forced to stop due to symptoms of LSS [17]. The SPWT is presented as a feasible and reproducible criterion measure for use with LSS and NC patients [18,19]. This was used for known groups comparison.

### Statistical analysis

Several statistical tests were used to establish the psychometric properties of the NCOS as follows:

Reliability: To test reliability the internal consistency of the questionnaire was measured using the Cronbach's alpha coefficient and alpha equal to or greater than 0.70 was considered satisfactory [20].

Validity: we used two types of validity. 1) Known groups comparison (discriminant validity): it was carried out to test how well the questionnaire discriminates between sub-groups of patients who differed in claudication distance as measured by the SPWT. The SPWT was extracted from patients’ case records as identified by surgeons and based on patients’ Oswestry Disability Index (ODI) categorized as poor, fair, good, and very good. It was hypothesized that patients with better condition would have higher score on the NCOS at preoperative assessment. One-way analysis of variance was performed to test the hypothesis. 2) Criterion validity (convergent validity): The correlation between changes on the NCOS and the ODI was assessed using the Pearson’s correlation coefficient and values of 0.40 or above were considered satisfactory (r ≥ 0.81-1.0 as excellent, 0.61- 0.80 very good, 0.41-0.60 good, 0.21-0.40 fair, and 0.0-0.20 poor) [20].

Responsiveness to change: Internal responsiveness to change as a psychometric property of the questionnaire also was assessed. It was operationalized as significant changes in patients’ scores due to the clinical intervention (surgery). As such patients’ pre- and post-operation scores were compared using the paired samples t-test in order to examine whether the NCOS was able to detect the significant changes following the clinical intervention (surgery) [21].

### Ethics

All patients gave their informed consent after receiving both written and oral information about the project. The Ethics Committee of Shahid Beheshti University of Medical Sciences, Tehran, Iran, approved the study protocol.

## Results

The characteristics of the NC patients and their scores on the NCOS are shown in Table 1. The mean age of patients was 61 (SD = 11) years; most were married (79%), and had completed primary or secondary education (67%). Almost all patients (99%) found the Iranian version of the NCOS acceptable. The mean NCOS score at preoperative and postoperative assessments were 26.9 (SD = 12.7) and 69.7 (SD = 16.8) respectively. With regard to morphology, 56 patients were classified as grade C stenosis and 28 patients as grade D stenosis.

**Table 1:** The characteristics of the study sample (n =84)

**Age groups (Year)**		**No.**	**%**
	30-54	23	27
	55-64	24	29
	≥65	37	44
	Mean (SD)	61 (11)	
	Range	30-84	
**Gender**			
	Male	25	30
	Female	59	70
**Educational status**			
	Illiterate	20	24
	Primary	44	52
	Secondary	12	15
	College/university	8	9
**Marital status**			
	Single	7	8
	Married	66	79
	Divorced/widowed	11	13
Morphology of the Dural Sac on MRI*			
	C	56	67
	D	28	33
NCOS score**			
Preoperative			
	Mean (SD)	26.9 (12.7)	
	Range (SD)	0-61	
Postoperative			
	Mean (SD)	69.7 (16.8)	
	Range	31-100	
ODI***			
Preoperative			
	Mean (SD)	32.4 (12.1)	
	Range	21-50	
Postoperative			
	Mean (SD)	14.9 (11.2)	
	Range	0-26	

* D: Extreme and C: Severity of lumbar spinal stenosis based on the morphology of the dural sac as observed on T2 axial MRI. There were no less severe groups in the study.

**. Higher scores on the NCOS indicate better conditions.

*** Higher scores on the ODI indicate worst conditions.

Reliability was assessed using the internal consistency of the questionnaire as measured by the Cronbach's alpha coefficient. The Cronbach's alpha coefficients were found to be 0.77 and 0.91 at pre- and post-operation assessments respectively, indicating a satisfactory internal consistency for the questionnaire.

Validity of the NCOS was examined using the known groups comparison. The preoperative NCOS discriminated well between sub-groups of patients who differed in walking distance as measured by the SWPT (P < 0.0001). The results are shown in Table 2. In addition, the change in the ODI after surgery was strongly correlated with the change in the NCOS, lending support to its good convergent validity (r = 0.81; P < 0.001).

**Table 2:** The preoperative NCOS by claudication distance among the study sample (known groups comparison)

**Claudication distance as measured by the SPWT**	Mean NCOS score*	**SD**
Poor (less than 100 meter)	19.9	9.9
Fair (between 100 and 800 meter)	24.8	12.4
Good (between 800 and 1600 meter)	28.9	13.7
Very Good (more than 1600 meter)	33.9	14.8
P**	< 0.0001	

* Higher scores indicate better conditions.

** Derived from one-way analysis of variance (the Bonferroni correction was used as post-hoc analysis).

Internal responsiveness to change was assessed by using paired samples t-test. In all instances the NCOS was responsive to the clinical intervention (surgery). In fact, the results indicated that patients’ functionality was significantly improved following surgery and the questionnaire was able to specify these improvements as expected. The results are shown in Table 3.

**Table 3:** Internal responsiveness of the NCOS to the clinical intervention (surgery)*

	**Preoperative**	**Postoperative**	P**
	**Mean (SD)**	**Mean (SD)**	
1. How far can you walk before having to stop and rest?	1.4 (0.4)	4.1 (0.5)	< 0.0001
2. How long can you stand still before having to sit down?	1.3 (0.2)	4.3 (0.7)	< 0.0001
3.Once your symptoms arise, you have:(back pain, leg pain, numbness/tingling, heaviness/weakness)	5.7 (1.4)	15.4 (2.6)	< 0.0001
4.The symptoms affect the following activities (sports or activities, household or odd jobs, walking, standing, sitting, sex life)	8.6 (4.1)	24.2 (2.4)	< 0.0001
5.How long must you rest before the symptoms resolve	1.6 (0.9)	4.6 (0.8)	< 0.0001
6.How frequently do you take pain medicine for these symptoms?	2.1 (0.5)	4.6 (0.4)	< 0.0001
7.How frequently do you see a doctor for these symptoms?	2.3 (0.8)	4.3 (0.9)	< 0.0001
8.Rank your pain on the VAS scale	3.9 (2)	8.2 (1.1)	< 0.0001
**Total**	26.9 (12.7)	69.7 (16.8)	< 0.0001

Scoring: The score is calculated by adding: ‘a’ answers = 0 points, ‘b’ answers = 2 points, ‘c’ answers = 4 points, ‘d’ answers = 6 points plus the pain scale added as 10-X.

Total possible points = 100 (asymptomatic, full function).

* Higher scores indicate better conditions.

** Derived from paired samples t-test.

## Discussion

The results of the present study showed that the Persian version of the NCOS is a reliable and valid instrument to measure functional status in patients with neurogenic claudication. However, since all patients in this study were grade C and grade D stenosis, the performance of this outcome measure in less severe groups of patients was not evaluated. In addition, one should be aware that in the current study due to special cultural circumstances (e.g., use of International System of Units vs. British system of measurement) and linguistic characteristics of Persian patients, some modifications were made. For instance in the original version walking distances are described in terms of miles or yards but we changed these to meter to make it understandable for Persian speaking patients.

The Cronbach’s alpha coefficients of the Persian NCOS were 0.77 and 0.91 at pre- and post-operation assessments, respectively. In order to compare our results with other similar studies, unfortunately we could not find any other studies reporting on the psychometric properties of the NCOS. However, the findings from the current study suggest that the Persian version of the questionnaire has a satisfactory internal consistency.

To the best knowledge of the authors, the Persian version of NCOS is the only condition-specific outcome measure for lumbar spinal stenosis patients with NC that has undergone psychometric evaluation in Iran. In addition, as far as we know, this article is the first report of an attempt to translate and validate the NCOS, because it has not been translated and validated for other languages and cultures. In general, this instrument seems to be a reliable and valid measure of outcome for assessing the functional status in patients with NC in Iran and perhaps it could be validated in other languages so that the results of possible up coming studies could be compared.

We used the walking distance as a measure for known groups comparison. The findings showed that the NCOS could discriminate well between sub-groups of patients who differed in the SWPT at preoperative assessment as expected. Thus it might be help full to use such a measure for similar studies in the future.

We carried out only a limited number of tests for this validation study. In future it might be necessary to perform other tests to establish stronger psychometric indexes for the NCOS. As such using recognized clinical measures for known group comparison is recommended. Perhaps performing factor analysis also might help to establish further psychometric evidence for the questionnaire. However, our main recommendation is to use simple and straightforward procedures to make technical issues understandable for clinicians as we did in the present study. Finally, it should be noted that the questionnaire was administered by a resident (PA) and thus we can only assume the results apply to an orally administered questionnaire.

## Conclusion

The findings from this preliminary validation study indicate that the Iranian version of the NCOS is a reliable and valid instrument for measuring functionality in patients with lumbar spinal stenosis who are suffering from neurogenic claudication.

## Competing interests

The authors declare that they have no competing interests.

## Authors’ contributions

PA was the main investigator and involved in the study design, data collection and writing process. HRM contributed to recruitment of patients, and provided additional data for this version of the manuscript. AM analyzed the data, critically evaluated the paper, responded to the reviewer’s comments and provided the final manuscript. All authors read and approved the final manuscript.

## Pre-publication history

The pre-publication history for this paper can be accessed here:

http://www.biomedcentral.com/1471-2377/12/101/prepub
